# What Our Contemporaries Think of Us

**Published:** 1895-09

**Authors:** 


### What Our Contemporaries Think of Us.

WE CANNOT resist the temptation to preserve this string of pearls. Nothing in our whole life has been so gratifying as this record of the esteem in which we are held by our contemporaries, and we take this opportunity to thank them, one and all, for their kind words and good wishes.

[Boston Medical and Surgical Journal.]

SEMI-CENTENNIAL ANNIVERSARY OF THE BUFFALO MEDICAL JOURNAL. —This excellent contemporary proposes to signalise its semi-centennial anniversary, which will occur in a few weeks, by increasing the number of its pages from sixty-four to eighty, and making other improvements.

[New York Medical Journal.]

Medical journals, like wine and violins, are prone to grow better as they grow older, and the BUFFALO MEDICAL JOURNAL shows manifest evidence that it conforms to the rule in this respect. We wish it centuries of ample prosperity.

[The Canadian Medical Review, Toronto.]

The BUFFALO MEDICAL JOURNAL, which was founded by the late Dr. Austin Flint, will be fifty years old in a few weeks. Its semi-centennial anniversary will be signalised by increasing its reading pages from sixty-four to eighty pages. There are very few medical journals that have reached this age, but this journal will never grow old or out of date. We extend our congratulations and best wishes for its continued prosperity.

[Leonard's Illustrated Medical Journal, Detroit.]

The BUFFALO MEDICAL JOURNAL is now close upon its fiftieth year. This will be celebrated in a fitting manner by an increase of good things between its covers. There are few, very few, medical journals reaching this lusty age. Success to its editor, and may more happy journal-birthdays come to him and his.

[The Buffalo Commercial.]

In a few weeks the BUFFALO MEDICAL JOURNAL will celebrate its semi-centennial anniversary. It is one of a very few medical publications which have attained the age of fifty years.

[The Buffalo Courier.]

The BUFFALO MEDICAL JOURNAL will be fifty years old in a few weeks. There are few medical journals in the country of equal age and if the medical brotherhood in Buffalo plays a more important part in the educational, philanthropic and social life of the community, as we think, than is often the case in our cities, it is perhaps due in part to the long existence here of a standard professional publication which has served to strengthen the *esprit de corps* and hold the members up to a high mark.

---

[The Journal of the American Medical Association.]

After a half century of good work in the cause of medical literature and for the welfare of mankind, the BUFFALO MEDICAL JOURNAL proposes to signalise its semi-centennial anniversary by increasing its reading pages and by making other improvements. The editors will please accept the congratulations of the *Journal of the American Medical Association*, on the youthful vigor which still characterises our Buffalo contemporary despite the fifty winters that have passed away since it was founded. We hope that our contemporary may still be young and as active when it celebrates its centenary.

---

[The Chicago Medical Recorder.]

The BUFFALO MEDICAL JOURNAL is fifty years old. Its semi-centennial will be signalised by increasing its reading pages to eighty and by other improvements. The journal is to be congratulated upon turning the half century mark. in which distinction it stands almost alone.

---

[The Philadelphia Polyclinic.]

There are few medical journals in this country that have reached the mature and dignified age of the BUFFALO MEDICAL JOURNAL and we heartily congratulate our contemporary, wishing that its future career may be even more prosperous and honored than its course hitherto.

---

[Maryland Medical Journal, Baltimore.]

The BUFFALO MEDICAL JOURNAL will celebrate, in a few weeks, its semi-centennial. This journal was founded by the late Dr. Austin Flint, and has always stood in the front rank of medical journalism.

---

[Virginia Medical Monthly, Richmond.]

The BUFFALO MEDICAL JOURNAL is about to celebrate its semi-centennial anniversary by increasing its reading pages from sixty-four to eighty and by other improvements. It is an excellent journal and deserves the success its announcement indicates it has achieved.

---

[The Cleveland Medical Gazette.]

We congratulate our esteemed contemporary, the BUFFALO MEDICAL JOURNAL, upon completing its half century of existence.

[Fort Wayne Medical Magazine.]

The BUFFALO MEDICAL JOURNAL was established in 1845 by the renowned Dr. Austin Flint and for fifty years has continued uninterrupted in its career of usefulness. We congratulate the present able and enterprising editors upon the success attained by it and predict a marked increase in its popularity and standing when the contemplated new changes shall have been inaugurated.

[Medical News, Philadelphia.]

The BUFFALO MEDICAL JOURNAL will signalise its approaching semi-centennial anniversary by increasing its reading pages and by making other improvements. It belongs in the class of medical publications that reflect honor and credit on the profession.

[Cincinnati Lancet-Clinic.]

The BUFFALO MEDICAL JOURNAL is well along in its fiftieth year and full of vim, vigor and vitality. There are not more than a half-dozen American medical journals that have turned the half-century point.

[College and Clinical Record, Philadelphia.]

The BUFFALO MEDICAL JOURNAL will be fifty years old in a few weeks. There are very few medical journals in this country that have reached the age of fifty years, and none that have exceled this valued contemporaneous publication in its fidelity to the best interests of the profession.

[Columbus Medical Journal.]

We desire to congratulate our friend and contemporary, Dr. Wm. Warren Potter, managing editor of the BUFFALO MEDICAL JOURNAL, on the near approach of its semi-centennial anniversary. There are few medical journals in this country that have reached the age of two score and ten.

[Texas Medical Journal, Austin.]

The BUFFALO MEDICAL JOURNAL will celebrate its semi-centennial shortly, by increasing its reading pages from sixty-four to eighty and making other improvements. The *Journal* extends its congratulations to Brother Potter ; but we want it distinctly understood that he has not been editing the JOURNAL all these years. Long may he and the JOURNAL live and prosper.

[The Quarterly-Journal of Inebriety, Hartford.]

The BUFFALO MEDICAL JOURNAL has passed the half-century milestone of existence, and is a typical example of the survival of the fittest. With Dr. Potter at the helm, one can safely predict another half century, free from rocks and storms, and replete with strong helpful influences for science and humanity.

[North Carolina Medical Journal, Wilmington.]

The BUFFALO MEDICAL JOURNAL will soon reach the ripe age of fifty years. This journal is already one of the very best medical monthlies in the country, and we extend our sincerest congratulations to the editor on the great success the past has brought, and wish him still more in the future.

---

[Louisville Medical Monthly.]

We congratulate Dr. Wm. Warren Potter on the fiftieth anniversary of his most excellent publication, the BUFFALO MEDICAL JOURNAL. But few journals in this country can claim such deserved distinction.

---

[Medical Mirror, St. Louis.]

The sterling monthly magazine, the BUFFALO MEDICAL JOURNAL, will have its fiftieth birthday this month, and its managing editor will signalise the event by increasing its reading pages and making many other improvements. Dr. Wm. Warren Potter is to be congratulated on the possession of so good a journal, one of the best in the United States. We should be glad to see every doctor in the United States a subscriber to it.

---

[Alabama Medical and Surgical Age.]

We congratulate the editor of the BUFFALO MEDICAL JOURNAL for the splendid success which his journal has achieved. Many journals during these hard times have gone to the wall, and it is an indication of much merit, as well as appreciation on the part of the profession, that the JOURNAL is enabled to increase the number of its reading pages and make other improvements.

---

[The Ophthalmic Record.]

The BUFFALO MEDICAL JOURNAL is now fifty years old. It will enter on the work of the last half of its first century enlarged and otherwise improved. Our friend, Dr. A. A. Hubbell, has been made an associate editor, which insures that the ophthalmic part of the JOURNAL will be kept up to date. Altogether it is one of the best monthlies published.

---

[The Medical Age, Detroit, Mich.]

Our esteemed contemporary, the BUFFALO MEDICAL JOURNAL, will be fifty years old in a few weeks, and its editor proposes to signalise its semi-centennial by increasing its reading pages and making other improvements that will keep it abreast of the progress of the period.

---

[The Atlantic Medical Weekly.]

The BUFEALO MEDICAL JOURNAL will be fifty years old in a few weeks. It is one of our most valued exchanges and we trust it may live to celebrate its centennial anniversary, and that in the next fifty years it may continue, as it has in the past, to work for the best interests of its large clientage, and for the advancement, improvement and upbuilding of the profession we all love so well.

---

[The Medical Examiner, New York.]

The BUFFALO MEDICAL JOURNAL has signalised its semi-centennial anniversary by increasing its reading pages and making several other improvements. It is one of the few medical periodicals that have attained the age of fifty years, and it has our congratulations upon this occasion.

---

[The Railway Surgeon, Chicago.]

The BUFFALO MEDICAL JOURNAL proposes to celebrate its semi-centennial (for it will be fifty years old in a few weeks) by increasing its size to eighty pages of reading matter and by making other improvements, which will make it an even more excellent journal than it has been heretofore.

---

[The Medical Herald, St. Joseph, Mo.]

The BUFFALO MEDICAL JOURNAL will celebrate its semi-centennial anniversary by increasing its reading matter to eighty pages and otherwise enhancing its value to its subscribers. We congratulate Dr. Potter upon the well-deserved success he has achieved in medical journalism.

---

[American Journal of Insanity, Chicago.]

The BUFFALO MEDICAL JOURNAL reaches its semi-centennial this year, being thus one of the veteran medical publications of this country. The *Journal*, as its senior by a year or more, takes this occasion of offering its editorial congratulations.

---

[The Canadian Practitioner, Toronto.]

The BUFFALO MEDICAL JOURNAL was first published in 1845 and will be fifty years old in a few weeks. It has for a long time been generally recognised as one of the best American medical monthly magazines. It scarcely needed anything in the way of improvement, and consequently the new efforts in that direction are all the more creditable because there had been no demand for them. Success to Dr. Potter and his fifty-year-old medical journal.

---

[Buffalo Sunday News.]

The BUFFALO MEDICAL JOURNAL, one of the best-known, most authoritative and widely quoted journals of its kind in the world, is approaching its half century birthday, and in August will appear in a new dress and will be fittingly illustrated to celebrate the event. The men who have conducted the journal have given to it an individuality seldom achieved by what are known as trade newspapers, and it has been a success of the most brilliant character.

---

[Medical and Surgical Reporter.]

The BUFFALO MEDICAL JOURNAL will be fifty years old in a few weeks. There are very few medical journals in this country that have reached the age of fifty years, and the *Reporter* extends its heartiest congratulations and cordial good-will to a contemporary which, after fifty years, has not passed the prime of life, but with renewed spirit promises to be still young when it rounds out its century.

---

[Colorado Climatologist and Denver Medical News, Denver, Col.]

Few among the many medical journals can count fifty years' existence, but the BUFFALO MEDICAL JOURNAL rounds the fiftieth mile post hale and hearty with an increase from sixty-four to eighty pages of reading matter. May more years—more success—more laurels, be yours, Brother Potter.

---

[Lehigh Valley Medical Magazine.]

How will we appear forty-four years hence ? if, indeed, there should be other than a back number on some upper shelf of a public library to look at. This apparently trival question is suggested by the statement that the youthful-looking and ever-welcome BUFFALO MEDICAL JOURNAL will be fifty years old this summer. If the years shall sit as lightly on us in 1939 as they do today on our Buffalo contemporary, and our appearance be as comely, we shall improve in vigor and in beauty with the passing years. We tender hosts of congratulations ; may your centennial find you hale and hearty.

---

[The Sanitarian, Brooklyn.]

The BUFFALO MEDICAL JOURNAL signalises its semi-centennial anniversary by a process which is alike gratifying to its readers and encouraging to its contemporaries—a mark of merited success on which *The Sanitarian* offers hearty congratulations.

---

[International Journal of Surgery, New York.]

The BUFFALO MEDICAL JOURNAL will celebrate in a few weeks its semi-centennial, and will signalise the occasion by increasing the number of its pages from sixty-four to eighty and make other improvements. It was founded by the late Dr. Austin Flint and has always stood in the front rank of medical journalism.

[Medical Record, New York.]

The BUFFALO MEDICAL JOURNAL is fifty years old. We extend our congratulations to our esteemed contemporary, and hope it will live to celebrate many more semi-centennials.

---

[Notes on New Pharmaceutical Products, St. Louis.]

The BUFFALO MEDICAL JOURNAL has, during all of its long and honored career, been a recognised authority in medical literature and it is but just to say that the tiara it wears has gained additional luster from the forceful, virile, editorial management imparted to it by Dr. Potter. Dignified, progressive, earnest and fearless are the typical adjectives marking his work and it gives ample assurance that the opening of its second semi-centennial presages even a more glorious fifty years than that now closed.

---

[The Atlanta Clinic.]

The BUFFALO MEDICAL JOURNAL was established in 1845 by Dr. Austin Flint, and has enjoyed this very unusual period of success. Dr. Wm. Warren Potter, the present managing editor, is one of the most forcible and interesting writers in the medical profession and whatever he contributes to medical literature possesses the genuine ring of worth. We congratulate our esteemed periodical on its half century of usefulness, and trust it may live, thrive and prosper for many such periods with unabating progress.

---

[St. Louis Medical and Surgical Journal.]

The BUFFALO MEDICAL JOURNAL has arrived to a good old age, hale and vigorous, and as aggressive as ever. It seems to partake of the nature of wine which improves with age. May all of us live to celebrate its next semi-centennial anniversary—if not in the flesh, in the spirit at least.

---

[Brooklyn Medical Journal.]

The BUFFALO MEDICAL JOURNAL is among the most highly prized of our exchanges, and we know of no better wish than we can make in behalf of its readers, than that in the fifty years to come it may be as ably edited as it has been in those just passed.

---

[Medical Bulletin, Philadelphia.]

Not many of the existing journals in the United States have enjoyed a continuous existence of fifty years. Among this number is the BUFFALO MEDICAL JOURNAL, which was founded in 1845. The career of this periodical has been honorable and successful. It has been and is edited with ability, has published instructive papers by many eminent men, is an influential professional medium and an exchange which is always consulted with pleasure and profit. It has recently announced two excellent additions to its editorial staff in the persons of Dr. Ernest Wende, clinical professor of dermatology in Buffalo University Medical College and health commissioner of Buffalo, and Dr. A. A. Hubbell, professor of diseases of the eye and ear in Niagara University Medical College. The best wishes of the editor of the *Medical Bulletin* are extended to its contemporary, in the hope and belief that its prosperity will be as signal in the future as in the past.

---

[The Medical Standard, Chicago, Ill.]

The BUFFALO MEDICAL JOURNAL has completed its half century and will celebrate the event by an increase in its reading pages. It has always been a credit to American medical journalism.

---

[Ontario Medical Journal, Toronto.]

In the middle of this month the BUFFALO MEDICAL JOURNAL was fifty years old, never having missed an issue since its inauguration. It is certainly beyond our province to criticise a confrère, but we may say that its large number of subscribers and long life should testify sufficiently of its worth without any of us expressing an opinion. Our congratulations are extended and we hope to be alive to receive return ones from them when our jubilee is celebrated.

---

[Western Reserve Medical Journal, Cleveland.]

The BUFFALO MEDICAL JOURNAL will this month celebrate its semicentennial anniversary. It is one of the oldest medical journals in the United States, and is deserving of great credit for the high journalistic tone which it has maintained during all these years. The celebration will be emphasised by an increase in the size of the journal and other improvements which will meet with the approval of its readers. It is so seldom that we have the opportunity of congratulating a contemporary upon arriving at the years of discretion that it becomes a pleasure for the *Western Reserve Medical Journal* to extend its most sincere congratulations to our Buffalo contemporary upon its arrival at the fiftieth year of its life, and it is our wish that the journal may live and prosper for fifty years more at least.

---

[The North American Medical Review, Kansas City.]

The BUFFALO MEDICAL JOURNAL will soon celebrate its semi-centennial anniversary by increasing its reading pages from sixty-four to eighty and making other improvements such as will keep it abreast of professional progress. Few medical journals in America have seen fifty years roll by, and the *Review* extends hearty congratulations and best wishes for its future.

---

[Health, Sanitation and Climatology of the United States, Washington.]

In 1839, the writer's father was a pupil of Dr. Joseph H. Flint, then a practising physician of Springfield, Mass., father to the founder of the BUFFALO MEDICAL JOURNAL and grandfather to the present distinguished physiologist in New York city. He was indebted to the Fints for many courtesies. Their example, suggestions and instruction served him in many instances in after years while engaged in practice. It is, therefore, a pleasure to note the continued success of a medical journal established by Austin Flint in 1845. May its present editors succeed in impressing their personality upon the American medical public as successfully and effectually as did their distinguished predecessor, and may the BUFFALO MEDICAL JOURNAL live as long as will the fame of Austin Flint.

---

[Ohio Medical Journal, Cincinnati.]

The BUFFALO MEDICAL JOURNAL, one of the ablest periodicals reaching this office, will, in a few weeks, celebrate its fiftieth anniversary. It will mark the event by increasing the number of its reading pages from sixty-four to eighty each issue. We heartily congratulate the editors, Drs. Thomas Lothrop and Wm. Warren Potter, both upon the journal's long successful career and upon its excellence.

---

[St. Louis Medical Era.]

It occasions us unusual pleasure to congratulate the BUFFALO MEDICAL JOURNAL on its approaching semi-centennial anniversary. It is not often that such an opportunity occurs, and, therefore, our appreciation of the event is particularly emphasised, especially as the journal which has attained such a distinction is still marked by all the attributes of vigorous life. When a journal reaches across half a century of time it must have achieved success, and its usefulness for the future is greatly increased.

---

[The Southern Clinic.]

The BUFFALO MEDICAL JOURNAL is fifty years old, and celebrated its semi-centennial anniversary by increasing its reading pages from sixty-four to eighty and other improvements of like value. Dr. Wm. Warren Potter, the worthy editor, has our most hearty congratulations on the occasion and our best wishes for his continued success.

---

[Clinique, St. Louis.]

The BUFFALO MEDICAL JOURNAL has reached its fiftieth anniversary. There are few medical journals in the country that have reached this age and it speaks well for the management.

[National Popular Review, San Diego.]

We join heartily in congratulating our esteemed contemporary, the BUFFALO MEDICAL JOURNAL, upon its having attained its fiftieth year of life. There are but few journals in medicine that can claim that longevity distinction ; and that our confrère can do so speaks volumes for the moral course it must have run in its youth as well as for the rectitude of its prime. With age it seemingly has gained rather than lost, as in its jubilee year it dons a new and youthful garb and increases its reading-matter by the addition of sixteen new pages. We wish our enterprising contemporary as long and as useful an existence as it has just passed.

---

[The General Practitioner, St. Louis.]

The BUFFALO MEDICAL JOURNAL, established by Austin Flint, M. D., in 1845, is about to celebrate its "golden wedding," as it were. We congratulate the editors on the length of its earthly pilgrimage, and hope the financial success of the JOURNAL has kept pace with its literary prosperity. The period of fifty years in medicine covers great changes in the lives and thoughts of men. In that time many truths have been discovered and many errors ruled for their little day and sunk into oblivion whence they were born, but the Great Watcher, who has separated the worthy from the unworthy, must have some way passed His approval on a medical journal that is allowed to maintain its existence for half a century.

---

[Hall's Journal of Health.]

The BUFFALO MEDICAL JOURNAL is about to celebrate its fiftieth birthday, an event that rarely occurs in the annals of medical journalism. The issue will be a large one in commemoration of the event, and the JOURNAL will be increased sixteen pages. There is no better journal published.

---

[Atlanta Medical and Surgical Journal.]

The BUFFALO MEDICAL JOURNAL is about to celebrate its semi-centennial. It was established fifty years ago by the late Austin Flint when there were only about a dozen journals in the country. Its career has been one of continuous prosperity and success. Under the able management of Dr. William Warren Potter it begins its second fifty years increased in size. We offer our best wishes for another half century of prosperity.

---

[Cincinnati Medical Journal.]

We extend to our venerable and respected contemporary, the BUFFALO MEDICAL JOURNAL, our sincere congratulations. That a medical journal or periodical of any kind lives for fifty years is proof positive that it is worthy of an existence and wide recognition, that it has been ably managed, and is an earnest that in the future it will increase, not only in size, but in worth, even according to the policy already mapped out by Dr. Potter.

---

[Denver Medical Times.]

The BUFFALO MEDICAL JOURNAL will celebrate its fiftieth anniversary with its August issue, at the same time increasing its number of pages from sixty-four to eighty. It has always kept thoroughly abreast with the professional progress of the period. We do not recall at present another medical journal which has been in existence fifty years. *The Denver Medical Times* will soon celebrate its fifteenth anniversary, and we were beginning to feel quite old until we learned of the aged Buffalo journal.

---

[Nashville Journal of Medicine and Surgery.]

We extend the heartiest congratulations to our esteemed contemporary, the BUFFALO MEDICAL JOURNAL, on the near approach of its semi-centennial anniversary. The *Nashville Journal* is six years its junior and we hope we can celebrate our semi-centennial in an equally substantial manner. We congratulate you, Dr. Potter, and earnestly hope that your JOURNAL will live to celebrate its centennial anniversary.

---

[New York Medical Times.]

The BUFFALO MEDICAL JOURNAL will celebrate its semi-centennial by increasing its reading pages and by other improvements. There are not many medical journals in our country which have reached the age of fifty years, and no better evidence of usefulness and popularity could be suggested. The *Times* extends its congratulations and best wishes and hopes that the JOURNAL may continue as long as it deserves.

---

[The Refractionist.]

The BUFFALO MEDICAL JOURNAL soon celebrates its golden wedding by increasing its reading matter from sixty-four to eighty pages and by making other improvements that will tend to keep it abreast of the times. The ophthalmological department is well looked after by Dr. Hubbell.

---

[National Medical Review, Washington, D. C.]

Showers of gold. May this be the case with the editor of the BUFFALO MEDICAL JOURNAL, who now celebrates the semi-centennial anniversary of his monthly. If he will only wait until this journal is fifty years of age we will promise him a present suitable to the occasion. As it is we can only congratulate him ahead for the showers of gold he is sure to receive from old subscribers and new.

[Annales D'Oculistique.]

The BUFFALO MEDICAL JOURNAL, which has long been a familiar and welcome visitor to a large circle of readers, is presently to celebrate its semi-centennial anniversary. The JOURNAL was one of the first to give to *Annales D' Oculistique* in its new form a hearty welcome, and we sincerely reciprocate its kindly sentiments. Its past history is abundant assurance that it will start on its second half-century with new vigor and interest.

---

[The North American Medical Review, Kansas City.]

The BUFFALO MEDICAL JOURNAL has reached its semi-centennial and has increased in interest *pari-passu.*

---

[Journal of Medicine and Science, Portland, Me.]

One of our best exchanges is the BUFFALO MEDICAL JOURNAL, which was established by Austin Flint in 1845 and which, therefore, is one of the oldest journals of the country. Its editors, Drs. Lothrop and Potter, propose to enlarge the issue which begins the second half century, have it written by physicians of Buffalo and devoted to the medical profession of that progressive and enterprising city. Dr. Potter, one of the editors referred to, was elected president of the National Confederation of State Medical Examining and Licensing Boards at its last meeting held during the recent meeting of the American Medical Association at Baltimore. Its object is to establish a uniform schedule of requirements for all medical colleges and examining boards, and to assist in perfecting the methods of higher medical education.

---

[Woman's Medical Journal, Toledo, O.]

A well rounded life of fifty years of activity is granted to but few of us, yet our valued exchange, the BUFFALO MEDICAL JOURNAL, has attained it and seems to have learned the lesson of living so well that the indications are excellent for another half century of existence. Judging the future by the past, our esteemed friend will enter the mystic circle of the twentieth century with a publication of such value to us all, that its perpetuity is assured.

---

[Buffalo Druggist.]

The jubilee number of the BUFFALO MEDICAL JOURNAL, established in 1845 by Austin Flint, M. D., is a remarkably creditable and interesting number. It is profusely illustrated with half-tone portraits of former editors of its columns and professors of the Buffalo and Niagara Medical Colleges, several of whom have achieved a world-wide reputation. In addition to a large amount of valuable professional reading matter, the number contains an exceedingly interesting special article, entitled 1845

—Then and Now—1895. The JOURNAL starts out on its second half century with every augury of continued prosperity and usefulness.

---

[Illustrated Buffalo Express, August 18, 1895.]

A half-century mark. The August number of the BUFFALO MEDICAL JOURNAL completes the fiftieth year of that publication. An article in the current number, entitled 1845--Then and Now—1895, by Dr. Wm. Warren Potter, is a history of the JOURNAL and of the vicissitudes and progress of medical journalism in Buffalo ; of the medical colleges, hospitals and medical societies. It is charmingly written—a true reminiscence, full and adequate as to data, graphic and entertaining. It is enriched with many portraits and other illustrations, and will long be conspicuous for its value among the historical monographs and essays of our home region. It is not often that the secular and the medical press have much to say to each other, or about each other. But this anniversary, and the conspicuous excellence of the work with which the JOURNAL has marked it, make it not only becoming, but a source of pleasure to *The Express*, to offer heartiest congratulations to our contemporary. Fame abides with the memory of the men who founded it; nor are her favors all for the past, for the day of greater things is at hand.

---

[Medical News.]

The BUFFALO MEDICAL JOURNAL has celebrated its fiftieth anniversary by the issue of a special edition of 128 pages, profusely illustrated, and containing some eighteen original articles, together with a historic review covering the period of its existence, and the usual editorial and other matter.

---

[The Canadian Practitioner.]

The jubilee number of the BUFFALO MEDICAL JOURNAL, published this month, reflects much credit on both publishers and editors. As we informed our readers in our issue for April, this journal is fifty years old, having been established in 1845 by the late Austin Flint, M. D. It was the first journal established between New York and Cincinnati or St. Louis, and proved a success in all respects in a very short time. The jubilee number contains a very interesting special article, written by Dr. William Warren Potter, giving the history of the JOURNAL for the fifty years, involving, as a matter of course, a history of the medical profession and medical colleges of Buffalo during that period. Again we extend to Dr. Potter our sincere congratulations and best wishes for continued success in the future.

[New York Medical Journal, August 24, 1895.]

The BUFFALO MEDICAL JOURNAL, having completed its fiftieth year, devotes a large part of the current number to an interesting account of its career up to the present time. At the present time, says Dr. Potter in the article referred to, it is the only medical journal published in the area bounded on the north by Toronto, on the east by Rochester, on the south by Pittsburg and on the west by Cleveland and Detroit. As a testimonial to the valued support that the JOURNAL has received from its contributors, subscribers and advertisers, says the writer, it now offers itself in an enlarged form and a new dress, for it well understands that it cannot hope to succeed without the continued favor of the medical profession, which it has enjoyed so long. This is the fiftieth year of its publication. While it is old in years, it must be young in activity, and it proposes to indicate its youthfulness by donning new garments, manifesting new energy, increasing the number of its pages and otherwise improving itself so as to make it worthy to stand in the front rank with the best medical journals in the land. In greater Buffalo there will be a greater BUFFALO MEDICAL JOURNAL. Dr. Potter also refers to the various medical colleges and hospitals, which have been established during the past fifty years in Buffalo. Many excellent illustrations are given and there are two views of the city of Buffalo, showing the marvelous growth that has taken place in the city during half a century. Dr. Potter says that he has tried to give a *résumé* of the salient events of that period, and in the illustrations he has sought to couple the past with the present, and, in some instances, to foreshadow the future.

---

[Indiana Medical Journal.]

The BUFFALO MEDICAL JOURNAL for August, 1895, is a jubilee number of 128 pages, and numerous half-tones, illustrating the growth of the city and JOURNAL for fifty years. The magazine was owned and edited by Austin Flint from 1845 to 1853, when he accepted the chair of practice in the University of Louisville. The special article, Then and Now, is by the present editor, Dr. Wm. Warren Potter, and is a complete and creditable account of fifty years of medical journalism and professional life in the western metropolis of New York state.

---

[Hot Springs Medical Journal.]

The jubilee number of the BUFFALO MEDICAL JOURNAL lies before us. It is a beautiful specimen of the printers' art and filled with first-class scientific material. It also gives us the pictures of the editorial workers on the JOURNAL from its foundation. Only one thing is lacking—the genial, warm-hearted and learned editor of today did not put his likeness along with Flint, Miner and Hunt. We wish he had done so.

